# Malaria in the postpartum period causes damage to the mammary gland

**DOI:** 10.1371/journal.pone.0258491

**Published:** 2021-10-13

**Authors:** Mamoru Niikura, Toshiyuki Fukutomi, Shoichiro Mineo, Jiro Mitobe, Fumie Kobayashi

**Affiliations:** 1 Department of Infectious Diseases, Kyorin University School of Medicine, Tokyo, Japan; 2 Department of Pharmacology and Toxicology, Kyorin University School of Medicine, Tokyo, Japan; 3 Department of Molecular Pathology, Tokyo Medical University, Tokyo, Japan; 4 Department of Environmental Science, School of Life and Environmental Science, Azabu University, Kanagawa, Japan; Universidade de Sao Paulo Instituto de Ciencias Biomedicas, BRAZIL

## Abstract

Mastitis is an inflammation of the mammary gland in the breast and is typically due to bacterial infection. In malaria-endemic areas, mastitis with accompanying fever can be challenging to differentiate from malaria. At the same time, it is unclear whether malaria infection is directly involved in the development of mastitis. In the present study, whether mastitis develops during infection with malaria parasites was investigated using a rodent malaria model with *Plasmodium berghei* (*P*. *berghei*; *Pb*) ANKA. The course of parasitemia in postpartum mice infected with *Pb* ANKA was similar to the course in infected virgin mice. However, infected postpartum mice died earlier than did infected virgin mice. In addition, the weight of pups from mice infected with *Pb* ANKA was significantly reduced compared with pups from uninfected mice. The macroscopic and histological analyses showed apparent changes, such as destruction of the alveolus wall and extensive presence of leukocytes, in mammary gland tissue in mice infected during the postpartum period. The findings suggest that women during the postpartum period are more vulnerable to complications when infected with malaria parasites, particularly women who do not acquire protective immunity against malaria parasites. Based on the proteomic analysis, IFN-γ signaling pathway-related proteins in mammary gland tissue of the infected postpartum mice were increased. Our results indicate that inflammation induced by IFN-γ, a proinflammatory cytokine, may contribute to negative histological changes in mammary gland tissue of postpartum mice infected with *Pb* ANKA. In IFN-γ receptor 1-deficient (IFNGR1-KO) mice, the histological changes in mammary gland tissue of the infected postpartum wild-type mice were improved to almost normal mammary gland structure. Furthermore, weight loss in pups delivered by infected IFNGR1-KO postpartum mice was not observed. Taken together, these findings indicate that inflammation induced by IFN-γ is associated with development of mastitis in postpartum mice infected with *Pb* ANKA. The present study results may increase our understanding of how disease aggravation occurs during postpartum malaria.

## Introduction

Malaria is caused by the genus *Plasmodium*, and it is the major parasitic disease in tropical and subtropical regions [1]. Five species of *Plasmodium* infect humans: *P*. *falciparum*, *P*. *vivax*, *P*. *malariae*, *P*. *ovale*, and *P*. *knowlesi*. Notably, *P*. *falciparum* causes severe pathologies such as cerebral and placental malaria in the blood stage. Children under the age of 5 years and pregnant women have the greatest risks of severe pathology during infection with *P*. *falciparum* [2–4].

The courses of cerebral and placental malaria involve proinflammatory cytokines [5, 6]. Toll-like receptors (TLRs) are pathogen-associated molecular patterns, which are involved in cerebral malaria and placental inflammation during malaria [7–9]. In a study using a mouse model, dendritic cells activated via TLR signaling expand pathogenic CD4^+^ T cells and CD8^+^ T cells, as well as the production of proinflammatory cytokines (e.g., IFN-γ) [10]. In addition to pathogenic CD4^+^ T cells and CD8^+^ T cells, macrophages and neutrophils (via IFN-γ receptor 1 [IFNGR1]) cause development of cerebral and placental malaria [11–14].

Pregnant women are highly susceptible to malaria infection, compared with nonpregnant women [15, 16]. Moreover, primigravid women are more susceptible to malaria infection than multigravid women [17]. The sequestration of VAR2CSA-expressing erythrocytes infected with malaria parasites and placental inflammation induced in women infected with malaria parasites have been associated with adverse pregnancy outcomes, such as fetal growth restriction, stillbirth, premature delivery, and (possibly) preeclampsia [5, 18–23]. In contrast to malaria during pregnancy, the risk of severe pathology is thought to decrease after delivery [24]. However, postpartum women reportedly have an increased risk of symptomatic infections, compared with nonpregnant women, in Africa [25, 26]. Mastitis, an inflammation of the mammary gland in the breast, is typically due to bacterial infection. Postpartum women often develop mastitis with accompanying fever [24]. In malaria-endemic areas, it can be challenging to differentiate mastitis and malaria [24]. At the same time, it is unclear whether malaria infection is directly involved in the development of mastitis.

Because a murine malaria model with *P*. *berghei* (*Pb*) ANKA shows features similar to human cerebral malaria (CM) and placental malaria [27–29], mice infected with *Pb* ANKA have been used to elucidate the pathogenesis of cerebral and placental malaria during infection with *P*. *falciparum* [10–13, 29]. *Pb* ANKA infection in mice might serve as an experimental model of malaria in postpartum period. In the present study, mastitis that developed during infection with malaria parasites was investigated using *Pb* ANKA. In order to understand the pathogenesis of postpartum malaria, pathological events of malaria during postpartum were compared with malaria during pregnancy.

## Materials and methods

### Animals and ethics

Female and male C57BL/6J (B6) mice (5–6 weeks of age) were purchased from CLEA Japan Inc. (Tokyo, Japan). IFN-γ R1-deficient (IFNGR1-KO) mice, which lack the receptor for IFN-γ [30], were purchased from Jackson Laboratories (Bar Harbor, ME, USA). The experiments were approved (#220) by the Experimental Animal Ethics Committee of Kyorin University School of Medicine, Tokyo, and all experimental animals were maintained in the animal facility in a specific pathogen-free unit with sterile bedding, food, and water. A female mouse > 9–15 weeks of age was mated for 1 day in a cage and examined for the presence of a vaginal plug the next morning.

The infection studies included frequent observations to determine humane endpoints, at which mice were unable to ambulate sufficiently to obtain water or food. At the indicated time points, mice were euthanized by cervical dislocation under inhalant anesthesia by isoflurane (n = 49). For bioluminescence analysis, mice were euthanized by cervical dislocation under 1.3 mg pentobarbital sodium anesthesia administered by intraperitoneal injection (n = 31). For comparative proteomic analysis, mice were euthanized by cervical dislocation (n = 18) and mammary gland tissues were removed. No mice died before they had met the criteria for euthanasia. All experiments were designed to minimize suffering. When illness or death was expected due to experimental infections, mice were visually checked by investigators at least twice daily (including weekends and holidays). Mice that exhibited signs of neurological distress, such as cerebral paralysis or depression, were immediately euthanized by cervical dislocation under isoflurane anesthesia and recorded as deaths (n = 67). All investigators who conducted the experiments had completed the Experimental Animal Ethics Committee training course on animal care and handling.

### Parasites and infections

Luciferase-expressing *Pb* ANKA were generated as previously described [31]. *Pb* ANKA were stored as frozen stocks in liquid nitrogen. *Pb* ANKA-infected erythrocytes were generated in donor mice inoculated intraperitoneally with a frozen stock of parasite. The donor mice were monitored for parasitemia daily and bled for experimental infection in ascending periods of parasitemia. To establish a mouse model of malaria in pregnancy, mice with or without a vaginal plug were injected intravenously with 1 × 10^4^ infected erythrocytes on day 12 post-mating because severe pathology occurs in the late phase of pregnancy [14, 29]. To establish a mouse model of malaria in the postpartum period, mice with or without a vaginal plug were injected intravenously with 1 × 10^4^ infected erythrocytes on day 7 postpartum because mammary gland tissue is mature on days 7 to 14 postpartum [32]. To induce reticulocytemia, blood (200 μL) was drawn from female mice (9–15 weeks of age). Mice were injected intravenously with 1 × 10^4^ infected erythrocytes on day 1 post-blood loss.

### Parasitemia and hematological changes

Methanol-fixed tail blood smears stained with 3% Giemsa diluted with phosphate buffer (pH 7.2) for 45 min were observed under a microscope. The number of infected erythrocytes in 250 erythrocytes was counted when parasitemia exceeded 10% and 1 × 10^4^ erythrocytes were examined when mice showed lower parasitemia. The percentage of parasitemia was calculated as follows: [(number of infected erythrocytes)/(total number of erythrocytes)] × 100.

To measure hematological changes, blood (200 μL) was obtained from mice and assessed using an automated hematology analyzer, Sysmex XT-2000i (SYSMEX Corp., Hyogo, Japan).

### Evaluation of experimental cerebral malaria

Female C57BL/6 (B6) mice on day 12 post-mating (pregnant), on day 7 post-delivery (postpartum), on day 1 post-blood loss (blood loss), and from age-matched virgin mice (virgin) were injected with 1 × 10^4^ erythrocytes that had been infected with *Pb* ANKA. Mice were checked twice daily. On days 7–8 post-infection, neurological signs, such as cerebral paralysis and depression, were monitored. After death, a surgical incision in the skin of the head was made and intracranial cerebral hemorrhage was confirmed in mice that developed experimental cerebral malaria (ECM) as previously described [33–35]. The virulence of *Pb* ANKA was defined based on ECM, mortality rate, and parasitemia.

### *Ex vivo* organ bioluminescence imaging

Bioluminescence imaging was performed using a Photon IMAGER system (Biospace Lab, Nesles-la-Vallée, France) as previously described [14]. Mice were administered 1.5 mg of VivoGlo™ Luciferin (In Vivo Grade) dissolved in 150 μL of phosphate buffered saline by intravenous injection. After mice had received the VivoGlo™ Luciferin, their organs were collected for image acquisition. A charge-coupled device camera was used to monitor the acquisition of emitted photons. *Ex vivo* bioluminescence imaging data were analyzed using the M3 software (Biospace) with size-constant regions of interest (ROIs).

### Histological examination of mammary gland tissue

Mammary gland tissues were obtained from uninfected and infected mice on day 7 post-infection. The mammary gland tissues were fixed in 10% buffered formalin and embedded in paraffin. Sections 6 μm thick were stained with hematoxylin and eosin (H&E) as previously described [35–37]. The stained sections were photographed at 20×, 100×, and 400× magnification using an All-in-One Fluorescence Microscope (BZ9000; KEYENCE Japan, Osaka, Japan).

### Comparative proteomic analysis

Proteins were extracted using Mammalian Protein Extraction Reagent (Thermo Fisher Scientific, Waltham, MA, USA) according to the manufacturer’s protocol and treated with trypsin. Comparative proteomic analyses were performed as previously described [33, 38]. All the fractionated peptides described above were injected into a trap column (C18, 0.3 × 5 mm; L-column, Chemicals Evaluation and Research Institute, Tokyo, Japan) and an analytical column (C18, 0.075 × 120 mm; Nikkyo Technos, Tokyo, Japan) attached to a nano liquid chromatography-tandem mass spectrometry (nanoLC-MS/MS) system. The nanoLC-MS/MS analysis was conducted using an LTQ Orbitrap Velos mass spectrometer (Thermo Fisher Scientific) equipped with a nanoLC interface (KYA, Tokyo, Japan) and a nano high-performance liquid chromatography (nanoHPLC) system (DiNa; KYA). Purified peptides from the nanoLC were introduced into the LTQ Orbitrap Velos, a hybrid ion-trap Fourier transform mass spectrometer. Full MS and MS/MS scans were followed by higher-energy collisionally activated dissociation (HCD). The database search engines Proteome Discoverer 1.4 (Thermo Fisher Scientific) and MASCOT 2.6 (Matrix Science) were used to identify and quantify proteins from the MS, MS/MS, and reporter ion spectra of peptides.

Peptide mass data were matched by searching the NCBInr database. The false discovery rate (FDR) [39] was calculated *via* peptide sequence analysis using Percolator [40]. High-confidence peptide identifications were obtained by setting a target FDR threshold of ≤ 1.0% at the peptide level. Proteins showing one or two peptide spectral matches (PSMs) were excluded. Protein levels were normalized to actin, cytoplasmic 1 (Accession: P60710), as previously described [33, 38]. Normalized experimental signal was calculated as follows: (observed experimental signal) ÷ (normalization factor).

### Statistical analysis

For time-series comparisons, Student’s *t*-test and one- and two-way ANOVAs with Fisher’s protected least significant difference (PLSD) post hoc test were performed using Statcel program (OMS, Saitama, Japan). Survival curves were compared using a log-rank test. *P*-values < 0.05 were considered statistically significant.

## Results

### Effects of pregnancy and the postpartum period on hematological parameters

To investigate hematological changes during pregnancy and the postpartum period, blood was obtained from uninfected female C57BL/6 (B6) mice on day 15 post-mating (pregnant), on day 10 post-delivery (postpartum), from virgin mice on day 1 post-blood loss (blood loss), and from age-matched virgin mice (control), then subjected to analysis (Tables 1 and 2). In pregnant mice, significant changes were not observed in white blood cells or platelets, compared with control mice (Table 1). Consistent with a previous report that reticulocytemia is induced by estrogen/ERα signaling during pregnancy [41], the proportion of reticulocytes in pregnant mice was significantly higher than in control mice (Table 2). Furthermore, increased neutrophils were observed in postpartum mice, compared with control, pregnant, and blood loss mice (Table 1). The proportion of reticulocytes was increased in postpartum mice, compared with control mice; however, their levels were lower than in pregnant mice (Table 2).

**Table 1:** Analyses of white blood cells (WBCs) and platelets (PLTs) in uninfected pregnant and postpartum mice.

	Control			Pregnant			Postpartum			Blood loss		
**WBC (μL)**	**8429.52**	**±**	**2552.32**	**6782.00**	**±**	**2253.73**	**7536.67**	**±**	**637.60**	**6845.56**	**±**	**2816.91**
**NEUT (μL)**	**702.86**	**±**	**299.45**	**602.50**	**±**	**118.99**	**1310.00**	**±**	255.14 *	**571.11**	**±**	**241.84**
**LYMPH (μL)**	**7191.43**	**±**	**2140.50**	**5126.00**	**±**	**1323.28**	**5713.33**	**±**	**421.47**	**5653.33**	**±**	**2408.92**
**MONO (μL)**	**413.33**	**±**	**207.06**	**604.00**	**±**	**242.96**	**376.67**	**±**	**180.09**	**522.22**	**±**	**204.19**
**EO (μL)**	**111.43**	**±**	**61.67**	**68.00**	**±**	**22.80**	**126.67**	**±**	**65.06**	**86.67**	**±**	**37.08**
**BASO (μL)**	**10.48**	**±**	**6.69**	**12.50**	**±**	**5.00**	**10.00**	**±**	**0.00**	**12.22**	**±**	**10.93**
**PLT (10** ^**3**^ **/μL)**	**955.00**	**±**	**286.93**	**1016.60**	**±**	**149.83**	**834.67**	**±**	**254.69**	**1198.67**	**±**	**163.46**

Blood was obtained from uninfected female C57BL/6 (B6) mice on day 15 post-mating (pregnant), on day 10 post-delivery (postpartum), from virgin mice on day 1 post-blood loss (blood loss), and from age-matched virgin mice (control). Experiments using three mice were performed in duplicate with similar results. Asterisks indicate a significant difference (*P* < 0.05 compared with control, pregnant, and blood loss; Tukey-Kramer and Dunnett tests). Neutrophil, NEUT; lymphocyte, LYMPH; monocyte, MONO; eosinophil, EO; basophil, BASO; platelet, PLT.

**Table 2:** Analyses of erythrocytes (RBCs) in uninfected pregnant and postpartum mice.

	Control			Pregnant			Postpartum			Blood loss		
**RBC (10** ^**4**^ **/μL)**	**1034.81**	**±**	**46.81**	**906.20**	**±**	38.49 *	**987.67**	**±**	**95.13**	**721.11**	**±**	54.32 *
**HGB (g/L)**	**156.38**	**±**	**4.49**	**136.80**	**±**	6.80 *	**150.67**	**±**	**11.37**	**111.89**	**±**	9.29 *
**HCT (%)**	**46.79**	**±**	**1.51**	**43.12**	**±**	**1.86**	**46.97**	**±**	**3.49**	**35.23**	**±**	3.17 *
**MCV (fL)**	**45.26**	**±**	**1.41**	**47.58**	**±**	0.30 *	**47.63**	**±**	1.66 *	**48.82**	**±**	1.61 *
**MCH (pg)**	**15.12**	**±**	**0.45**	**15.10**	**±**	**0.19**	**15.30**	**±**	**0.40**	**15.52**	**±**	**0.24**
**MCHC (g/L)**	**334.24**	**±**	**6.07**	**317.40**	**±**	5.123 *	**320.67**	**±**	2.89 *	**317.78**	**±**	6.36 *
**RET (%)**	**4.36**	**±**	**0.72**	**11.89**	**±**	1.90 *	**6.06**	**±**	0.62 *	**7.21**	**±**	1.00 *

Blood was obtained from uninfected female C57BL/6 (B6) mice on day 15 post-mating (pregnant), on day 10 post-delivery (postpartum), from virgin mice on day 1 post-blood loss (blood loss), and from age-matched virgin mice (control). Experiments using three mice were performed in duplicate with similar results. Asterisks indicate a significant difference (*P* < 0.05 compared with control; Tukey-Kramer and Dunnett tests). Red blood cell, RBC; hemoglobin, HGB; hematocrit, HCT; mean corpuscular volume, MCV; mean content in Hb, MCH; mean corpuscular Hb concentration, MCHC; reticulocyte, RET.

### Effects of hematological changes during pregnancy and the postpartum period on the outcome of infection with malaria parasites

**Fig 1:**
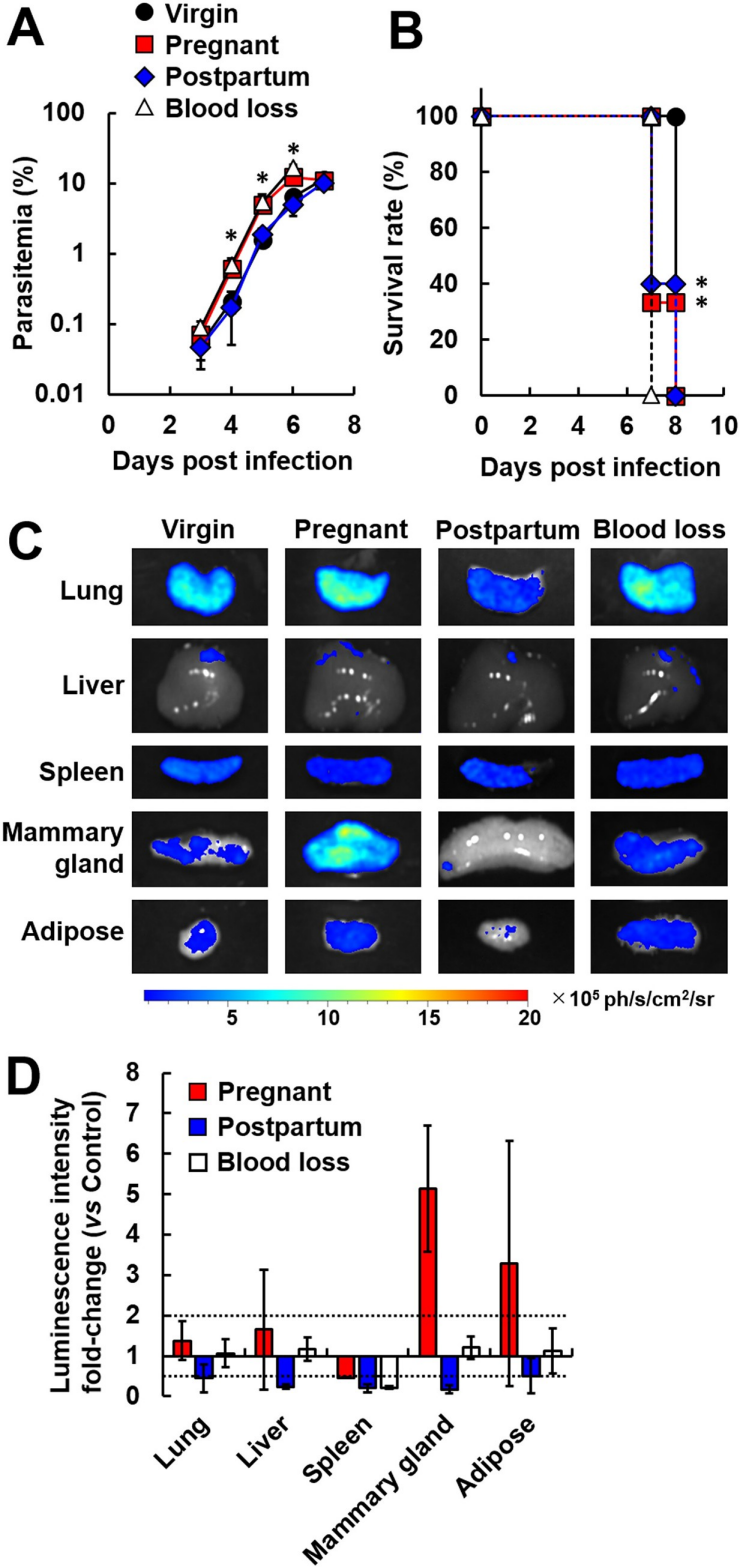
Comparative analysis of pregnant and postpartum mice infected with malaria parasites. Female C57BL/6 (B6) mice on day 12 post-mating (pregnant), on day 7 post-delivery (postpartum), on day 1 post-blood loss (blood loss), and age-matched virgin mice (virgin) were injected with 1 × 10^4^ infected erythrocytes of luciferase-expressing *Plasmodium berghei* (*Pb*) ANKA. (A) Course of parasitemia. (*P* < 0.05, compared with virgin and postpartum mice; Tukey-Kramer and Dunnett tests) (B) Survival rate. Asterisks indicate a significant difference (*P* < 0.05, compared with virgin mice; log-rank test). Results are expressed as means ± standard deviation (SD) of three mice. Experiments were performed in triplicate with similar results. (C) Bioluminescent images of luciferase activity in the organs of mice from each group. D-luciferin (1.5 mg) was injected into the tail vein of mice and the organs of mice from each group were removed on day 3 post-infection. Representative data are shown. (D) Fold change indicates the alteration of luciferase activity in each group, compared with virgin mice. Dotted lines indicate significant difference (≥ 2-fold or ≤ 0.5-fold). Results are expressed as means ± SD of three mice. Experiments were performed in duplicate with similar results.

To investigate the effects of hematological changes during pregnancy and the postpartum period on the outcome of infection with malaria parasites, pregnant and postpartum mice were infected with rodent malaria parasites, *Pb* ANKA. In a previous study, a rapid increase in parasitemia was observed in mice with reticulocytemia because *Pb* ANKA invades reticulocytes [42]. In this study, blood-loss mice were used as controls for reticulocytemia. Parasitemia in blood-loss mice infected with *Pb* ANKA rapidly increased, compared with infected virgin and postpartum mice, beginning on day 3 post-infection (Fig 1A). Similar to blood-loss mice, a rapid increase in parasitemia was observed in pregnant mice that had been infected with *Pb* ANKA (Fig 1A). Furthermore, infected pregnant mice died earlier than did infected virgin mice (Fig 1B), suggesting that reticulocytemia is also a risk factor for a rapid increase in parasitemia during malaria in pregnancy. Although postpartum mice showed reticulocytemia comparable to blood-loss mice, the course of parasitemia in postpartum mice infected with *Pb* ANKA was similar to the course in infected virgin mice (Fig 1A). However, postpartum mice infected with *Pb* ANKA developed ECM (data not shown) and died earlier than did infected virgin mice (on day 7 post-infection) (Fig 1B).

### Erythrocytes infected with *Pb* ANKA do not accumulate in the mammary gland in postpartum mice

Erythrocytes infected with malaria parasites bind to vascular endothelial cells, resulting in erythrocyte accumulation in organs, such as adipose tissue [43]. As the accumulation pattern of infected erythrocytes changes during pregnancy [14], we used bioluminescence imaging to investigate whether the accumulation pattern of infected erythrocytes is also altered during the postpartum period. Mice showing parasitemia of > 1% are not suitable for bioluminescence imaging because strong luciferase activity causes interference [14]. Therefore, organs were collected from mice showing parasitemia of < 0.1% (on day 3 post-infection). In infected pregnant mice, high luciferase activity levels in mammary gland tissue were observed, compared with control mice and infected postpartum mice (Fig 1C and 1D). By contrast, the luciferase activity levels in mammary gland tissue of infected postpartum mice were much lower than in control mice (Fig 1C and 1D).

Schizonts of malaria parasites bind to vascular endothelial cells by interacting with the endothelial receptor CD36 and sequestration-related proteins, such as SBP1 and MAHRP1a [43]. *Pb* ANKA lacking SBP1 or MAHRP1a exhibited reduced sequestration and parasitemia in mice [43]. To examine whether schizonts of *Pb* ANKA bind to vascular endothelial cells of organs, mice were infected with purified schizonts of *Pb* ANKA. During synchronized infection, pregnant mice showed high luciferase activity levels in the mammary gland, compared with control mice and infected postpartum mice, after perfusion (S1 Fig). By contrast, luciferase activity levels in mammary gland tissue of infected postpartum mice were lower than in control mice (S1 Fig). These results suggest that the accumulation of infected erythrocytes in the mammary gland was reduced during the postpartum period.

### Adverse effects on offspring of malaria-infected mice during the postpartum period

**Fig 2:**
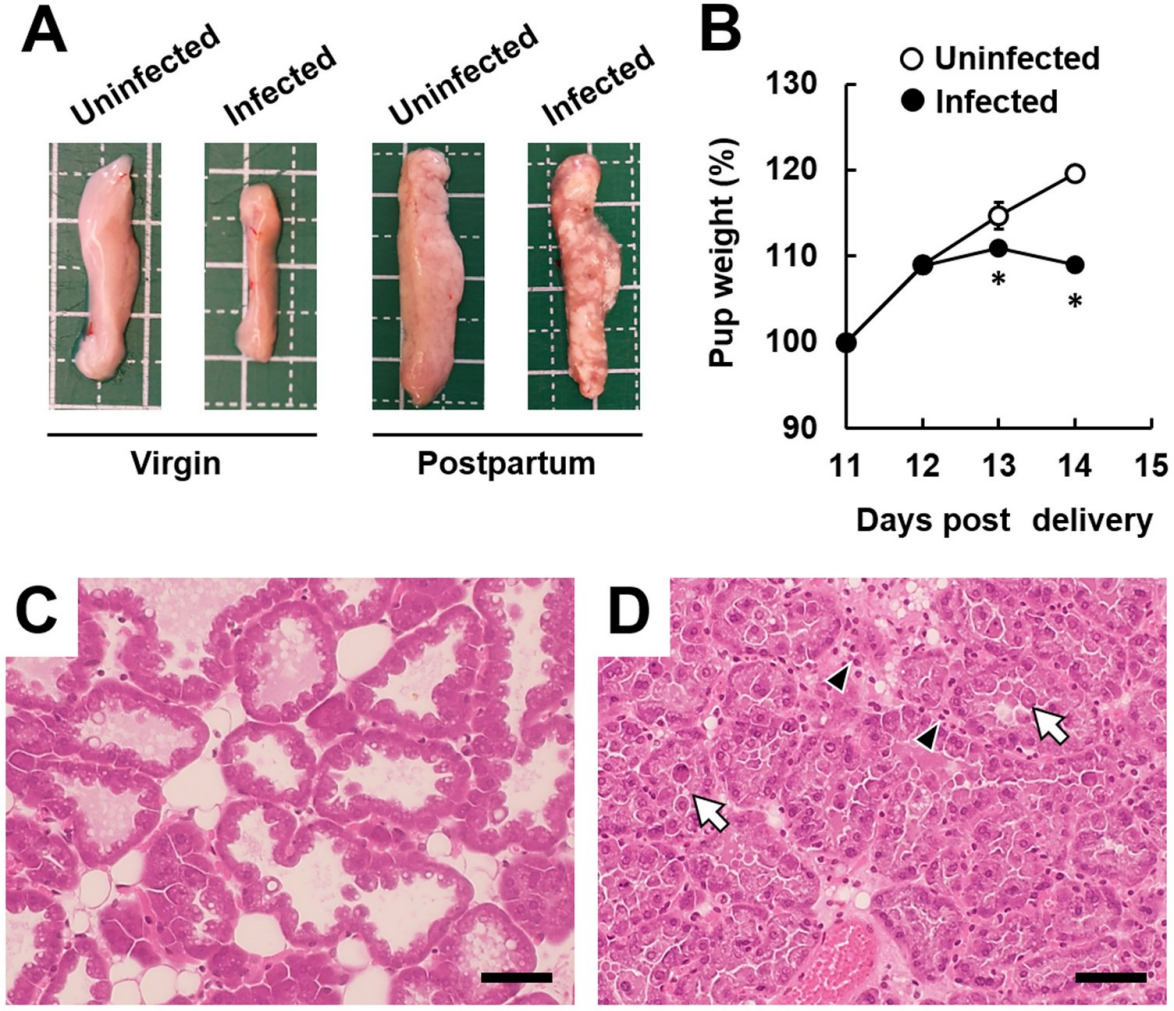
Development of mastitis in postpartum mice infected with malaria parasites. B6 mice on day 7 post-delivery were injected with 1 × 10^4^ infected erythrocytes of luciferase-expressing *Plasmodium berghei* (*Pb*) ANKA. (A) Macroscopic analysis of mammary gland tissue. Mammary gland tissues were removed on day 14 post-delivery (on day 7 post-infection). Experiments were performed in triplicate with similar results. Representative data are shown. (B) Pup weight. The weight is expressed as a percentage of the value on day 11 post-delivery. Results are expressed as means ± standard deviation (SD) of pups delivered by the mother. Experiments were performed in triplicate with similar results. Asterisks indicate a significant difference (*P* < 0.05, compared with pups delivered by uninfected mice; Student’s *t*-test). (C and D) Histological analyses of mammary gland tissue on day 14 post-delivery. (C) Uninfected postpartum mice. (D) Infected postpartum mice. Representative hematoxylin and eosin (H&E)-stained tissue sections are shown. The scale bar represents 50 μm. Arrows indicate destruction of an alveolus wall. Arrowheads indicate extensive presence of leukocytes. Experiments were performed in triplicate with similar results.

For analysis of ECM development, macroscopic analysis of organs, in addition to the brain, was performed. The results showed pathological alteration of mammary gland tissue, such as tissue atrophy and sporadic milk stasis, in infected postpartum mice (Fig 2A). Therefore, the pup weight was measured and histological analyses of mammary gland tissue were performed. The weight of pups delivered by infected postpartum mice was significantly reduced, compared with pups delivered by uninfected mice from day 13 post-delivery (on day 6 post-infection; Fig 2B). Histological analyses of mammary gland tissue were performed on day 14 post-delivery (on day 7 post-infection). Compared with the mammary gland tissue of uninfected postpartum mice, apparent histological changes were observed in the infected postpartum mice, represented by destruction of the alveolus wall and extensive presence of leukocytes (Fig 2C and 2D). In contrast, histological analyses revealed a few and immature mammary glands in pregnant mice, compared with postpartum mice (Fig 2C and 2D, S2C–S2F Fig). Substantial pathological changes were not observed in immature mammary gland tissue of infected pregnant mice (S2C–S2F Fig). These findings indicated that destruction of the alveolus wall in the mammary gland occurs after the pups’ delivery, in the postpartum period.

### Proteomic analysis of mammary gland tissue in postpartum mice infected with malaria parasites

**Fig 3:**
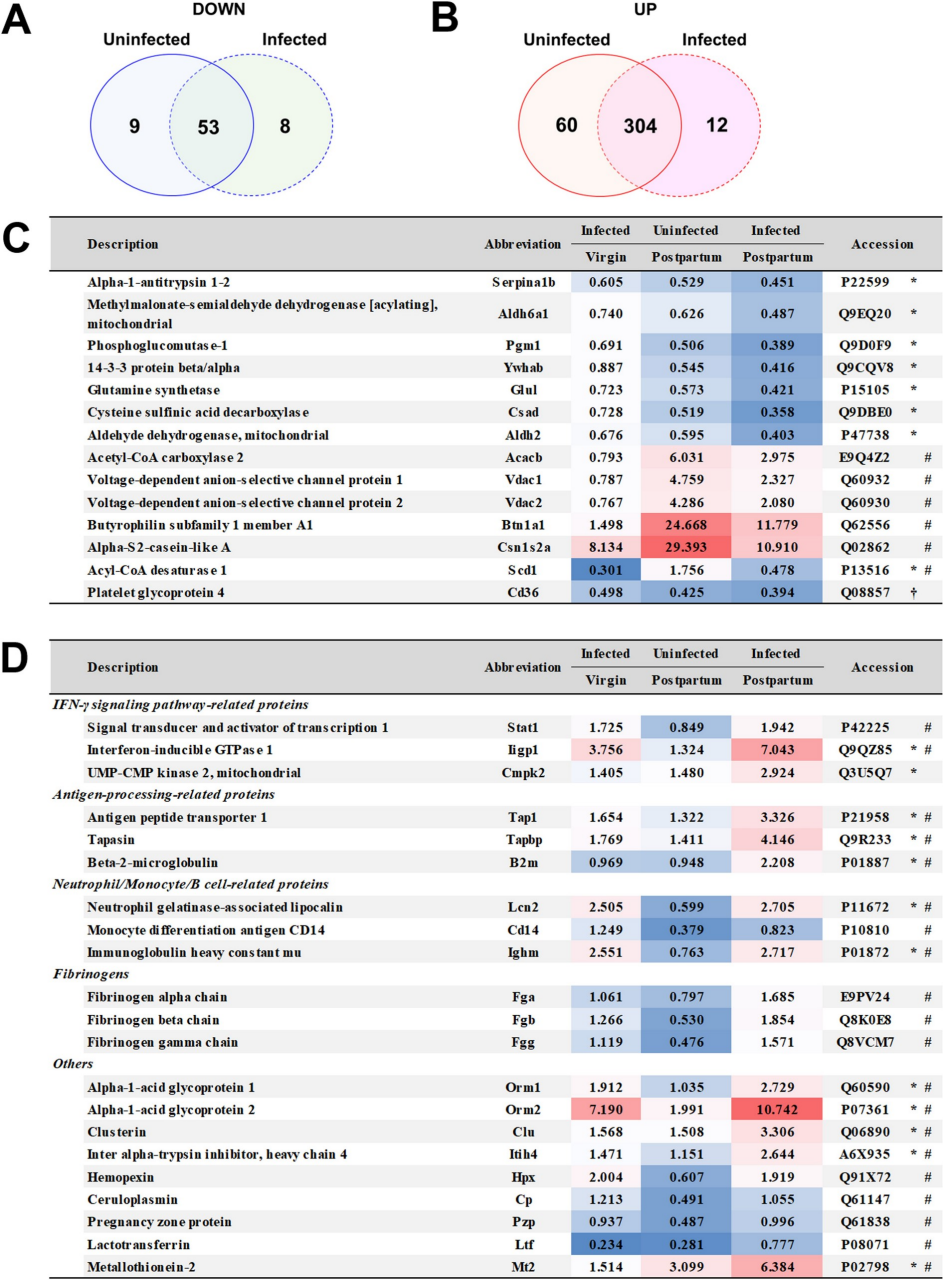
Effects of malaria on the proteome in mammary gland tissue of postpartum mice. (A and B) Venn diagram of protein levels in mammary gland tissue of uninfected and infected postpartum mice that changed 0.5-fold (A) or 2-fold (B) compared with uninfected virgin mice. (C and D) Fold change indicates the change in protein levels compared with uninfected virgin mice. (C) Protein levels that were decreased in mammary gland tissue of infected postpartum mice. Asterisks indicate proteins with significantly lower levels than in uninfected virgin mice (≤ 0.5-fold change). Hash marks indicate proteins with significantly lower levels than in uninfected postpartum mice (≤ 0.5-fold change). The dagger indicates notable proteins levels that were decreased in mammary gland tissue of uninfected and infected postpartum mice, compared with uninfected virgin mice. (D) Protein levels that were increased in mammary gland tissue of infected postpartum mice. Asterisks indicate proteins with significantly higher levels than in uninfected virgin mice (≥ 2-fold change). Hash marks indicate proteins with significantly higher levels than in uninfected postpartum mice (≥ 2-fold change). S1 Table shows all proteins detected in this study. Data are representative of three independent experiments.

To understand the initial events of development of mastitis in malaria during the postpartum period, we investigated the proteome of mammary gland tissues before substantial pathological changes became evident by comparative proteomic analysis. The proteome of mammary gland tissues in postpartum mice infected with *Pb* ANKA on day 6 post-infection was compared with the proteomes in uninfected virgin and postpartum mice, as well as infected virgin and postpartum mice (Fig 3 and S1 Table). In the comparative proteomic analysis, 1,100 proteins were detected. Among the 1,100 proteins, 715 proteins showing three or more PSMs were analyzed. The protein levels in malaria parasites were normalized to actin, cytoplasmic 1 (Accession: P60710). The levels of eight proteins in infected postpartum mice were markedly decreased, compared with uninfected virgin mice (Fig 3A). In addition, lipid metabolism-related proteins (e.g., Acetyl-CoA carboxylase 2 [Acacb], Acyl-CoA desaturase 1 [Scd1], and Butyrophilin subfamily 1 member A1 [Btn1a1]), mitochondrial and plasma membrane-related proteins (e.g., voltage-dependent anion-selective channel protein [Vdac] 1 and Vdac2), and alpha-S2-casein-like A (Csn1s2a; i.e., a milk protein) exhibited significantly lower levels in infected postpartum mice than in uninfected postpartum mice, indicating dysfunction of the mammary gland tissue (Fig 3C).

By contrast, the levels of 12 proteins, such as IFN-γ signaling pathway-related proteins, antigen-processing-related proteins, and neutrophil/monocyte/B cell-related proteins, in infected postpartum mice were significantly higher than in uninfected virgin and postpartum mice (Fig 3B and 3D). The levels of IFN-γ signaling pathway-related and antigen-processing-related proteins were increased in infected virgin mice but their levels were lower than in infected postpartum mice (Fig 3D). These results indicated that IFN-γ signaling may contribute to histological change in the mammary gland tissue in postpartum mice infected with malaria parasites.

We next examined the proteome in mammary gland tissues of infected pregnant mice. Mammary gland tissues were obtained from infected pregnant mice on day 5 post-infection because parasitemia in infected pregnant mice on day 5 was comparable with parasitemia in infected postpartum mice on day 6 post-infection (Fig 1A). Increased levels of IFN-γ signaling pathway-related proteins (e.g., signal transducer and activator of transcription 1 [Stat1] and interferon-inducible GTPase 1 [Iigp1]) and antigen-processing-related proteins (e.g., antigen peptide transporter 1 [Tap1] and Tapasin [Tapbp]) were observed in mammary gland tissue of infected pregnant mice (S3 Fig and S2 Table). However, the levels of Iigp1 and Tapbp in infected pregnant mice tended to be lower than in infected postpartum mice (Fig 3D and S3D Fig). In contrast to infected postpartum mice, neutrophil/monocyte/B cell-related proteins were not detected in mammary gland tissue of infected pregnant mice (S3 Fig and S2 Table).

### Effects of IFNGR1-deficiency on histological changes in mammary gland tissue in postpartum mice infected with malaria parasites

In our previous study, IFNGR1 signaling was shown to be involved in adverse pregnancy outcomes during infection with *Pb*NK65 [14]. To investigate whether the immune response to IFN-γ participates in adverse pregnancy outcomes during *Pb* ANKA infection, pregnant IFNGR1-KO mice were infected with *Pb* ANKA. Consequently, preterm delivery observed in infected pregnant wild-type mice on day 6 post-infection did not occur in infected pregnant IFNGR1-KO mice (S3 Table) as well as in *Pb*NK65-infected pregnant IFNGR1-KO mice.

**Fig 4:**
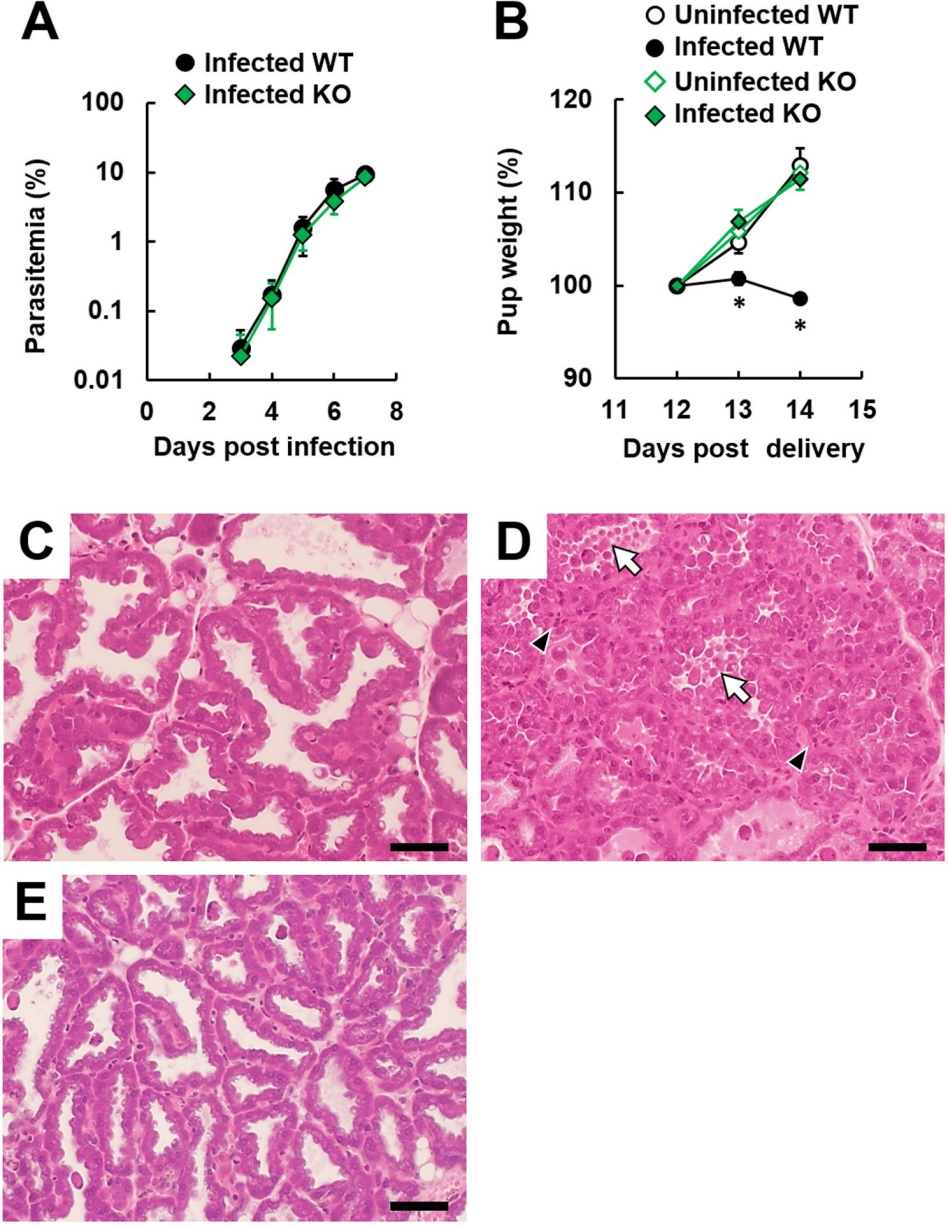
IFN-γ signaling-dependent histological change in the mammary gland tissue of postpartum mice infected with malaria parasites. Wild-type (WT) B6 mice and IFNGR1-KO mice (KO) on day 7 post-delivery were infected with 1 × 10^4^ infected erythrocytes of luciferase-expressing *Plasmodium berghei* (*Pb*) ANKA. Green symbols and lines indicate IFNGR1-KO mice. (A) Course of parasitemia. Results are expressed as means ± standard deviation (SD) of three mice. Experiments were performed in duplicate with similar results. (B) Pup weight. The weight is expressed as a percentage of the value on day 12 post-delivery. Open symbols represent weight of pups born from uninfected mice. Closed symbols indicate weight of pups born from infected mice. Results are expressed as means ± SD of pups delivered by the mother. Experiments were performed in triplicate with similar results. Asterisks indicate a significant difference (*P* < 0.05; Tukey-Kramer and Dunnett tests). (C–E) Histological analyses of mammary gland tissue on day 14 post-delivery. (C) Uninfected postpartum WT mice. (D) Infected postpartum WT mice. (E) Infected postpartum IFNGR1-KO mice. Representative hematoxylin and eosin (H&E)-stained placental sections are shown. The scale bar represents 50 μm. Arrows indicate destruction of an alveolus wall. Arrowheads indicate extensive presence of leukocytes. Experiments were performed in triplicate with similar results.

Whether the immune response to IFN-γ contributes to histological change in the mammary gland tissue during *Pb* ANKA infection remains unclear, therefore, histological change in the mammary gland tissue in postpartum mice infected with malaria parasites was investigated by infecting IFNGR1-KO postpartum mice with *Pb* ANKA. The course of parasitemia in IFNGR1-KO postpartum mice infected with *Pb* ANKA was similar to the course in infected wild-type postpartum mice (Fig 4A). However, weight loss in pups delivered by infected IFNGR1-KO postpartum mice was not observed (Fig 4B). Furthermore, histological changes observed in the mammary gland tissue of infected wild-type postpartum mice, such as destruction of the alveolus wall, hemorrhage, and the extensive presence of inflammatory cells, were improved in infected IFNGR1-KO postpartum mice on day 14 post-delivery (on day 7 post-infection; Fig 4C–4E). These findings indicated IFNGR1 signaling plays a pivotal role in pathological changes in mammary gland tissue and subsequent adverse effects on offspring during severe malaria.

## Discussion

In the present study, the effects of pregnancy and the postpartum period on the outcome of infection with malaria parasites were explored using a mouse model of malaria. Pregnant mice infected with *Pb* ANKA showed rapid increases in parasitemia and adverse pregnancy outcomes. In contrast, although the course of parasitemia in postpartum mice infected with *Pb* ANKA was similar to the course in control mice, postpartum mice infected with malaria parasites developed mastitis, resulting in significantly reduced pup weight, compared with pups delivered by uninfected mice. Moreover, the infected postpartum mice died earlier than did infected virgin mice. These findings indicated that the development of mastitis during infection with malaria parasites could occur during the postpartum period, particularly in women who do not acquire protective immunity against malaria parasites.

In the mammary gland tissue of postpartum mice infected with malaria parasites, infiltration of leukocytes was observed on histological analyses. These findings were confirmed with proteome data showing that neutrophil/monocyte/B cell-related proteins in the mammary gland tissue of infected postpartum mice were significantly higher than in uninfected virgin and uninfected postpartum mice. Through analyses of white blood cells in uninfected mice, we found that the neutrophil count in postpartum mice was higher than in control mice and pregnant mice. Neutrophil infiltration was observed in the mammary glands of bacteria-induced mastitis [44, 45]. These findings suggested that systemic inflammation induced by neutrophils may occur during malaria in the postpartum period. Notably, bioluminescence imaging revealed that the accumulation of infected erythrocytes in mammary gland tissue of infected postpartum mice was lower than the accumulation in control mice, suggesting that the accumulation of infected erythrocytes is not associated with mastitis during with infection with malaria parasites in the postpartum period.

The adult mammary gland undergoes dynamic changes during pregnancy and the postpartum period [46]. In mammary gland tissue of postpartum mice, mature mammary glands were increased, compared with tissue collected from pregnant mice. In this study, infected postpartum mice (but not infected pregnant mice) showed substantial pathological changes and dysfunction in mammary gland tissue. Comparative proteomic analysis revealed that the levels of IFN-γ signaling pathway-related proteins (but not neutrophil/monocyte/B cell-related proteins) in infected pregnant mice were higher than in infected virgin mice. Based on these results, mature mammary gland tissue of postpartum mice is presumably more prone to inflammation than immature mammary gland tissue of virgin mice and pregnant mice.

Increases in IFN-γ signaling pathway-related and antigen processing-related proteins were observed in the mammary gland tissue of infected postpartum mice. In IFNGR1-KO postpartum mice infected with *Pb* ANKA, substantial pathological changes in the mammary gland tissue were not observed. Furthermore, their offspring growth was comparable to offspring from uninfected mice. These results indicated that IFNGR1 signaling contributes to the development of mastitis during infection with malaria parasites.

Although the course of parasitemia in postpartum mice infected with *Pb* ANKA was similar to the course in infected virgin mice, infected postpartum mice died earlier than did infected virgin mice. Furthermore, development of mastitis occurred earlier than the death of mice infected with *Pb* ANKA in the present study. Excessive inflammation has been shown to cause the development of ECM and death of mice infected with *Pb* ANKA [11]. Based on our results, the inflammation induced postpartum in mammary gland tissue may contribute to the development of ECM and the early death of postpartum mice infected with *Pb* ANKA.

High luciferase activity levels in mammary gland tissue were observed in pregnant mice. Schizonts were detected in vessels around mammary glands. However, comparative proteomic analysis showed the CD36 protein level in mammary gland tissue of pregnant mice was comparable with virgin mice. Based on these results, the increased accumulation of infected erythrocytes in mammary gland tissue during pregnancy was apparently independent of CD36. In the immature mammary gland tissue of pregnant mice, high levels of leukemia inhibitory factor receptor and alpha-1B-glycoprotein, a membrane-related protein, were observed. These proteins may be involved in the accumulation of infected erythrocytes in immature mammary gland tissue of pregnant mice. By contrast, our results may help increase the sensitivity for detecting malaria during pregnancy.

Infectious mastitis is commonly caused by an infection of pathogenic microorganisms, such as *Staphylococcus* and *Streptococcus* [47]. Bacteria-induced mastitis is a local infection and involves local inflammation. In this study, the systemic inflammation caused by *Pb* ANKA infection is responsible for inducing mastitis in mice during the postpartum period and resulted in reduced pup weight. Breastfeeding provides nutrition and immune protection to babies. Therefore, careful attention should be given to postpartum malaria patients to prevent the spread of infectious diseases. However, the pathology of lactating women infected with malaria parasites has not been fully investigated. Our experimental findings indicated that additional investigation is required to establish whether lactating women infected with malaria parasites also develop mastitis and severe pathology. Further research is also needed to elucidate the detailed mechanism by which mastitis develops during malaria, including the sequestration of infected erythrocytes to mammary gland tissue and the role of immune cells, neutrophils, CD8^+^T cells, and IgA-secreting cells.

## Supporting information

S1 Fig Bioluminescent images of luciferase activity in the organs of infected mice. For bioluminescence analysis, erythrocytes infected with *Pb* ANKA were transferred to RPMI-1640 medium supplemented with 25% fetal bovine serum, 0.05 mg/mL penicillin, and 0.05 mg/mL streptomycin. Infected erythrocytes were incubated for 18 h in 90% N_2_, 5% CO_2_, and 5% O_2_. Mature schizonts and gametocytes were harvested by Nycodenz density gradient centrifugation [48]. Left panel, C57BL/6 (B6) mice on day 14 post-mating (pregnant), on day 10 post-delivery (postpartum), and age-matched virgin (virgin) mice were injected with 5 × 10^6^–5 × 10^7^ schizonts of luciferase-expressing *Plasmodium berghei* (*Pb*) ANKA parasites. At 22 h post-infection, D-luciferin (1.5 mg) was injected into the tail vein of mice and the organs of mice from each group removed after perfusion. Representative data are shown. Right panel, fold change indicates the change in luciferase activity in each group, compared with virgin mice. Dotted lines indicate significant difference (≥ 2-fold or ≤ 0.5-fold). Results are expressed as means ± standard deviation (SD) of three mice. Experiments were performed in duplicate with similar results.
(TIF)

S2 Fig Histological analyses of mammary gland tissue during pregnancy. Pregnant B6 mice on day on day 12 post-mating were injected with 1 × 10^4^ infected erythrocytes of *Plasmodium berghei* (*Pb*) ANKA. Representative hematoxylin and eosin (H&E)-stained placental sections are shown. (A and B) Uninfected wild-type virgin mouse. (C and D) Uninfected wild-type pregnant mouse on day 17 post-mating. (E and F) Infected wild-type pregnant mouse on day 17 post-mating (on day 5 post-infection). (G and H) Infected IFNGR1KO pregnant mouse on day 17 post-mating (on day 5 post-infection). (A, C, E, G) The scale bar represents 1,500 μm. (B, D, F, H) The scale bar represents 50 μm. (C, E, G) Numerous developing mammary glands were observed compared with A. Arrows indicate schizonts in vessels around mammary glands. Experiments were performed in triplicate with similar results.
(TIF)

S3 Fig Effects of malaria on the proteome in mammary gland tissue of pregnant mice. (A and B) Venn diagram of protein levels in mammary gland tissue of uninfected and infected pregnant mice that changed 0.5-fold (A) or 2-fold (B) compared with uninfected virgin mice. (C and D) Fold change indicates the change in protein levels compared with uninfected virgin mice. (C) Protein levels that were significantly decreased and unchanged in mammary gland tissue of infected pregnant mice. Asterisks indicate proteins with significantly lower levels than in uninfected virgin mice (≤ 0.5-fold change). Hash marks indicate proteins with significantly lower levels than in uninfected pregnant mice (≤ 0.5-fold change). The dagger indicates the notable protein levels that were comparable with mammary gland tissue of uninfected virgin mice. (D) Proteins levels that were significantly increased in mammary gland tissue of infected pregnant mice, compared with uninfected virgin mice (≥ 2-fold change). The IFN-γ signaling pathway-related proteins, antigen processing-related proteins, and other proteins obtained from the 59 increased proteins in mammary gland tissue of infected pregnant mice. Hash marks indicate proteins with significantly higher levels than in uninfected pregnant mice (≥ 2-fold change). S2 Table shows all protein detected in this study. Data are representative of two independent experiments.
(TIF)

S1 Table Comparative proteomic analysis of mammary gland tissue in uninfected or infected postpartum mice. The proteome of mammary gland tissues in postpartum mice infected with *Pb* ANKA on day 6 post-infection was compared with the proteomes in uninfected virgin and postpartum mice, as well as infected virgin and postpartum mice. Proteins showing one or two peptide spectral matches (PSMs) were excluded. Protein levels were normalized to actin, cytoplasmic 1 (Accession: P60710).
(XLSX)

S2 Table Comparative proteomic analysis of mammary gland tissue in uninfected or infected pregnant mice. The proteome of mammary gland tissues in pregnant mice infected with *Pb* ANKA on day 5 post-infection was compared with the proteomes in uninfected virgin and pregnant mice, as well as infected virgin and pregnant mice. Proteins showing one or two peptide spectral matches (PSMs) were excluded. Protein levels were normalized to actin, cytoplasmic 1 (Accession: P60710).
(XLSX)

S3 Table Pregnancy outcomes in pregnant mice infected with *Pb* ANKA. Mice on day 12 post-mating were injected with 1 × 10^4^ erythrocytes that had been infected with luciferase-expressing *Plasmodium berghei* (*Pb*) ANKA. The number, survival rate, and weight of pups were measured on day 0 post-delivery. Experiments using three mice were performed in duplicate with similar results. Asterisks indicate a significant difference (*P* < 0.05, compared with the pregnancy period of uninfected mice; Tukey-Kramer and Dunnett tests).
(XLSX)

## Abbreviation

*P*. *berghei*; *Pb*: *Plasmodium berghei*
ECM: Experimental cerebral malaria
IFNGR1KO: IFN-γ receptor 1-deficient
