# Supplementary figures and images for: Malaria in the postpartum period causes damage to the mammary gland
Source: PLoS One. 2021 Oct 13;16(10):e0258491. doi: 10.1371/journal.pone.0258491 (PMC8513860; doi:10.1371/journal.pone.0258491)

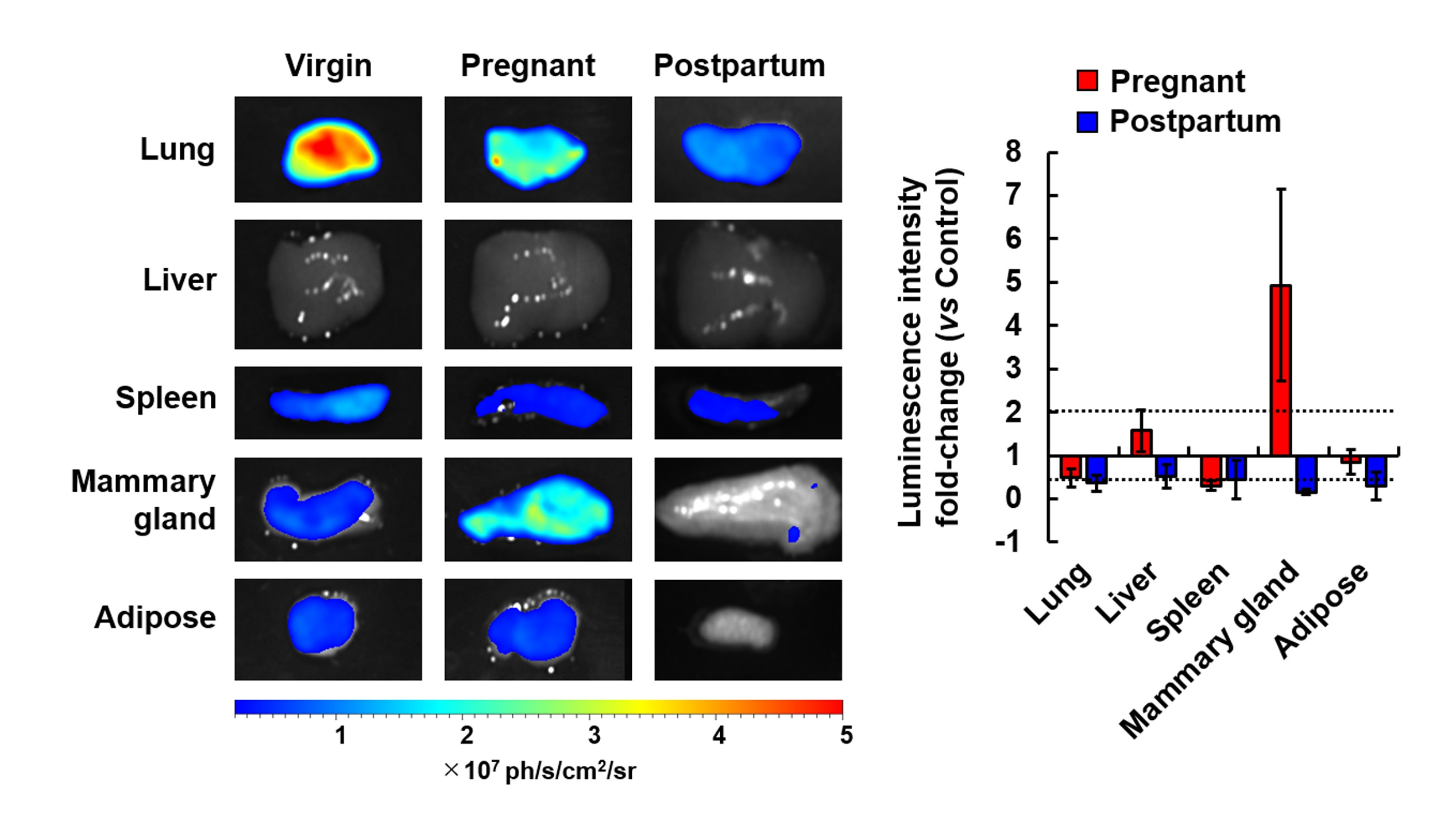

Supplement: S1 Fig — For bioluminescence analysis, erythrocytes infected with Pb ANKA were transferred to RPMI-1640 medium supplemented with 25% fetal bovine serum, 0.05 mg/mL penicillin, and 0.05 mg/mL streptomycin. Infected erythrocytes were incubated for 18 h in 90% N2, 5% CO2, and 5% O2. Mature schizonts and gametocytes were harvested by Nycodenz density gradient centrifugation [48]. Left panel, C57BL/6 (B6) mice on day 14 post-mating (pregnant), on day 10 post-delivery (postpartum), and age-matched virgin (virgin) mice were injected with 5 × 10^6^–5 × 10^7^ schizonts of luciferase-expressing Plasmodium berghei (Pb) ANKA parasites. At 22 h post-infection, D-luciferin (1.5 mg) was injected into the tail vein of mice and the organs of mice from each group removed after perfusion. Representative data are shown. Right panel, fold change indicates the change in luciferase activity in each group, compared with virgin mice. Dotted lines indicate significant difference (≥ 2-fold or ≤ 0.5-fold). Results are expressed as means ± standard deviation (SD) of three mice. Experiments were performed in duplicate with similar results. (TIF) [file pone.0258491.s001.tif]

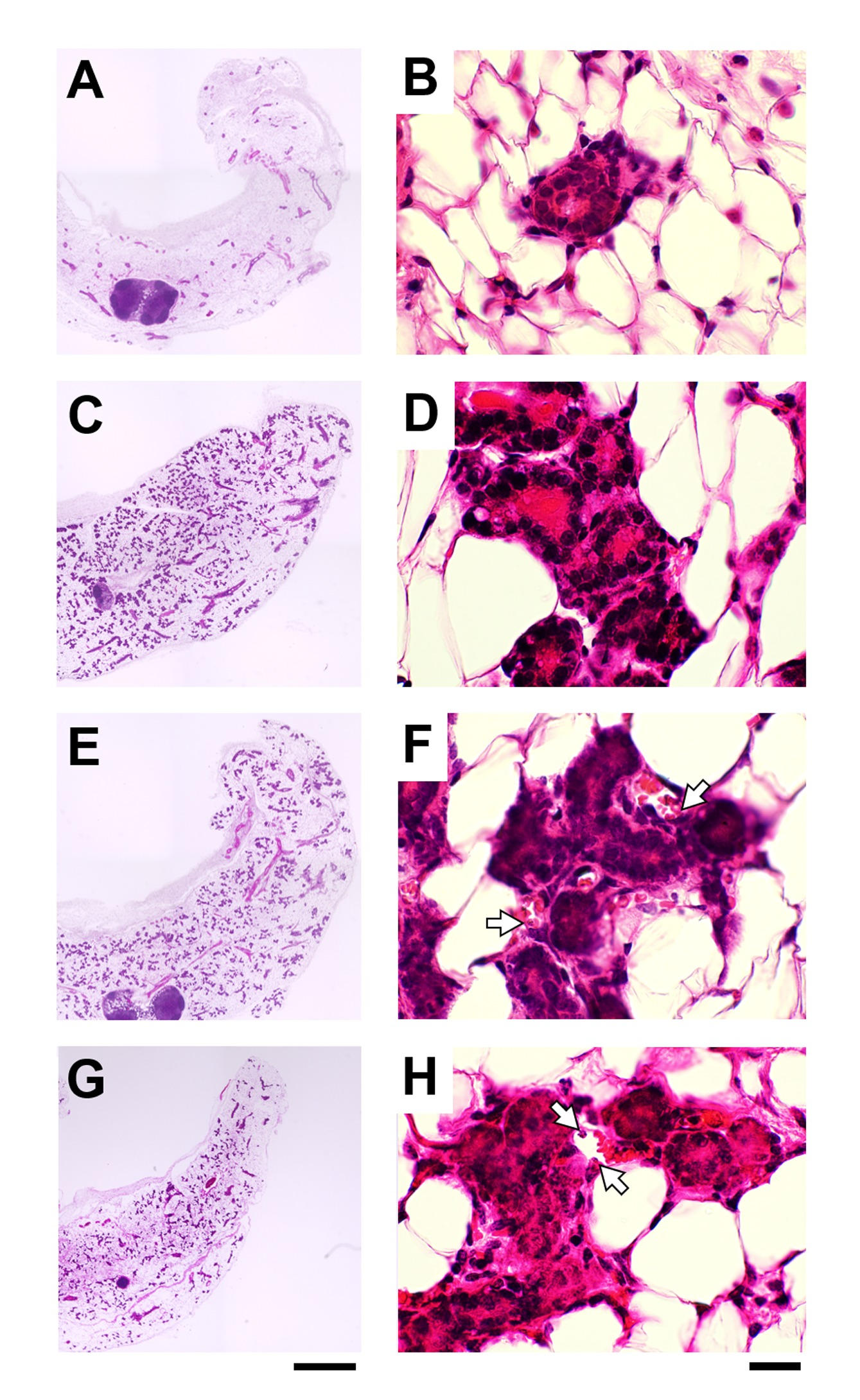

Supplement: S2 Fig — Pregnant B6 mice on day on day 12 post-mating were injected with 1 × 10^4^ infected erythrocytes of Plasmodium berghei (Pb) ANKA. Representative hematoxylin and eosin (H&E)-stained placental sections are shown. (A and B) Uninfected wild-type virgin mouse. (C and D) Uninfected wild-type pregnant mouse on day 17 post-mating. (E and F) Infected wild-type pregnant mouse on day 17 post-mating (on day 5 post-infection). (G and H) Infected IFNGR1KO pregnant mouse on day 17 post-mating (on day 5 post-infection). (A, C, E, G) The scale bar represents 1,500 μm. (B, D, F, H) The scale bar represents 50 μm. (C, E, G) Numerous developing mammary glands were observed compared with A. Arrows indicate schizonts in vessels around mammary glands. Experiments were performed in triplicate with similar results. (TIF) [file pone.0258491.s002.tif]

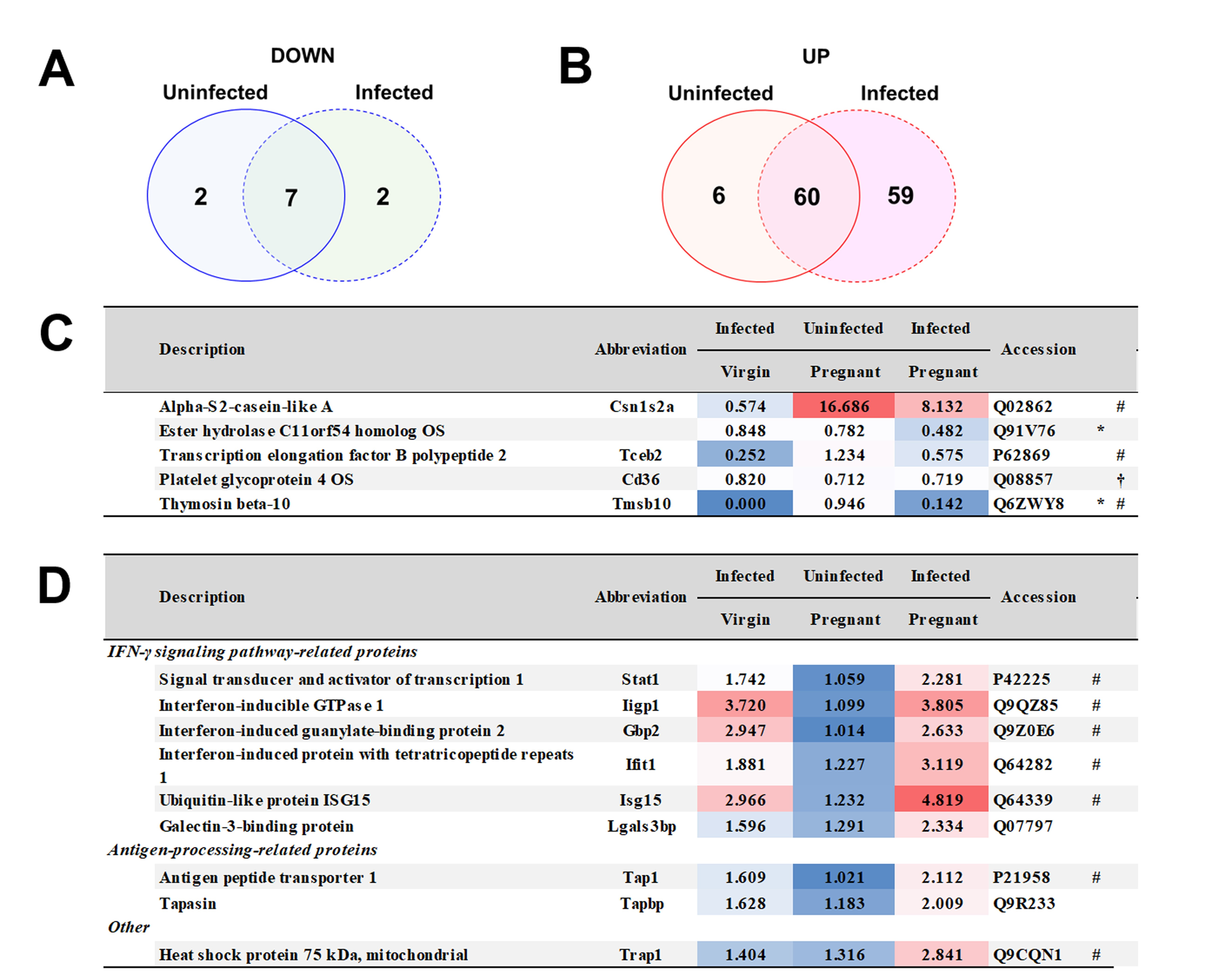

Supplement: S3 Fig — (A and B) Venn diagram of protein levels in mammary gland tissue of uninfected and infected pregnant mice that changed 0.5-fold (A) or 2-fold (B) compared with uninfected virgin mice. (C and D) Fold change indicates the change in protein levels compared with uninfected virgin mice. (C) Protein levels that were significantly decreased and unchanged in mammary gland tissue of infected pregnant mice. Asterisks indicate proteins with significantly lower levels than in uninfected virgin mice (≤ 0.5-fold change). Hash marks indicate proteins with significantly lower levels than in uninfected pregnant mice (≤ 0.5-fold change). The dagger indicates the notable protein levels that were comparable with mammary gland tissue of uninfected virgin mice. (D) Proteins levels that were significantly increased in mammary gland tissue of infected pregnant mice, compared with uninfected virgin mice (≥ 2-fold change). The IFN-γ signaling pathway-related proteins, antigen processing-related proteins, and other proteins obtained from the 59 increased proteins in mammary gland tissue of infected pregnant mice. Hash marks indicate proteins with significantly higher levels than in uninfected pregnant mice (≥ 2-fold change). S2 Table shows all protein detected in this study. Data are representative of two independent experiments. (TIF) [file pone.0258491.s003.tif]
